# Nanoparticles use for Delivering Ursolic Acid in Cancer Therapy: A Scoping Review

**DOI:** 10.3389/fphar.2021.787226

**Published:** 2021-12-24

**Authors:** Andang Miatmoko, Ester Adelia Mianing, Retno Sari, Esti Hendradi

**Affiliations:** ^1^ Department of Pharmaceutical Sciences, Faculty of Pharmacy, Universitas Airlangga, Surabaya, Indonesia; ^2^ Stem Cell Research and Development Center, Universitas Airlangga, Surabaya, Indonesia; ^3^ Study Program of Pharmacy, Faculty of Pharmacy, Universitas Airlangga, Surabaya, Indonesia

**Keywords:** ursolic acid, cancer, nanoparticle, liposome, nanosphere, polymeric micelle, efficacy, toxicity

## Abstract

Ursolic acid is a natural pentacyclic triterpenoid that exerts a potent anticancer effect. Furthermore, it is classified as a BCS class IV compound possessing low permeability and water solubility, consequently demonstrating limited bioavailability in addition to low therapeutic effectiveness. Nanoparticles are developed to modify the physical characteristics of drug and can often be produced in the range of 30–200 nm, providing highly effective cancer therapy due to the Enhanced Permeation and Retention (EPR) Effect. This study aims to provide a review of the efficacy and safety of various types of Ursolic Acid-loading nanoparticles within the setting of preclinical and clinical anticancer studies. This literature study used scoping review method, where the extracted data must comply with the journal inclusion criteria of within years of 2010–2020. The identification stage produced 237 suitable articles. Duplicate screening was then conducted followed by the initial selection of 18 articles that had been reviewed and extracted for data analysis. Based on this review, the use of nanoparticles can be seen to increase the anticancer efficacy of Ursolic Acid in terms of several parameters including pharmacokinetic data, survival rates and inhibition rates, as well as the absence of serious toxicity in preclinical and clinical trials in terms of several parameters including body weight, blood clinical chemistry, and organ histipathology. Based on this review, the use of nanoparticles has been able to increase the anticancer efficacy of Ursolic Acid, as well as show the absence of serious toxicity in preclinical and clinical trials. Evenmore, the liposome carrier provides development data that has reached the clinical trial phase I. The use of nanoparticle provides high potential for Ursolic Acid delivery in cancer therapy.

## Introduction

Cancer is a disease that can occur in almost any organ or tissue of the body when abnormal cells grow uncontrollably beyond their usual limits to invade adjacent parts of the body and/or spread to other organs (Kamińska et al., 2015). Data from the Global Burden of Cancer released by the World Health Organization (WHO) reported that the number of cases and deaths from cancer in 2018 totaled 18.1 million and 9.6 million respectively. Cancer-related deaths are predicted to increase to more than 13.1 million by 2030 (Internatioanl Agency for Research on Cancer, 2018). The most common types affecting men include lung, prostate, colorectal, and liver cancer, while in women, they comprise breast, colorectal, lung, cervical and thyroid cancers. In 2018, the incidence rate of liver cancer in Indonesia ranked eighth highest in Southeast Asia, while in Asia as a whole it occupied 23rd position with 348,809 cases (Globocan. Indonesia - Global Cancer Observatory WHO, 2018)*.*

The first-line options for cancer treatment include surgery, radiotherapy and the administering of biological and chemical drugs (chemotherapy, hormones, and biological therapy). However, such forms of treatment fail to control metastatic tumors that have spread to other organs (Kumari et al., 2016). Chemotherapeutic agents are predominantly toxic compounds that primarily inhibit the rapid proliferation of cancer cells, while also potentially restricting the growth of hair follicle, bone marrow and gastrointestinal cells culminating in severe undesired side effects (Pérez-Herrero and Fernández-Medarde, 2015). Consequently, the effect of certain natural compounds have been widely explored and it has been scientifically proven that Ursolic Acid (UA), an active agent, inhibits the proliferation of cancer cells (Kashyap et al., 2016).

UA is a natural pentacyclic triterpene of the cyclosqualenoid family present in many plants which can modulate cellular transcription factors, growth factor receptors, inflammatory cytokines, and numerous other molecular targets that regulate cell proliferation, metastasis, apoptosis, angiogenesis, and autophagy. The anticancer effects of UA have been reported for many types of cancer, one of which is liver cancer (Khwaza et al., 2020). The mechanisms of UA which produce such effects include nuclear-kappa B (NF-kB) factors and apoptosis signaling in cancer cells (Seo et al., 2018). The protease activity involving urokinase and cathepsin B, both of which are known to be associated with tumor invasion and metastasis, is also significantly inhibited by UA, interleukin-1 β (IL-1β), Tumor necrotic factor-α (TNF-α), and the expression of MMP-2 and MMP-9 (Mitochondria-Mediated Pathway) (Kashyap et al., 2016; Ali et al., 2019). Prolonged administration of excessive doses of UA, with an LD50 value of 9.26 g/kg in acute toxicity tests on mice (Sun et al., 2020), has the potential to cause liver cytotoxicity which is not classified as genetic toxicity. Within the Biopharmaceutical Classification System (BCS), UA is categorized as a BCS class IV compound demonstrating low permeability and solubility (Sun et al., 2020) which, consequently, requires a nanotechnology-based drug delivery system to reach the desired target (Biopharmaceutical, 2011). In particular, the development of drug delivery systems entails the use of nanoparticles targeted at cancer cells which significantly improve therapeutic and diagnostic efficacy as well as reducing unwanted side effects.

Nanotechnology represents a new therapeutic platform that employs nanoparticles (NPs) in the diagnosis and treatment of cancer. NPs are used in cancer therapy due to their relatively small size, 1–1,000 nm, the fact that they frequently fall within the range of 10–200 nm, and the presence of the EPR effect (Rajesh, 2000; Reddy, 2005; Fang et al., 2011). Nano-drug delivery systems have been lauded for their excellent biocompatibility properties, low toxicity, increased solubility in water, in addition to their ability to deliver targeted drugs to tissues which limits their accumulation in the kidneys, liver, spleen, and other non-targeted organs, while improving therapeutic efficacy (Kumari et al., 2016; Li et al., 2017). The delivery of nanoparticles to tumor tissues through systemic circulation can be executed through two targeting strategies, including passive targeting*,* when nanoparticles entering circulation will accumulate at the tumor site due to enhanced permeation and retention (EPR). In contrast, active targeting*,* generally employs ligand molecules such as antibodies and peptides to recognize specific antigens expressed in tumor cells or the microenvironment (Kumari et al., 2016).

Many types of nanoparticles exist, including polymeric therapy (polymer-protein and polymer-drug conjugates) in which drugs are covalently bound or conjugated to polymer structures and nanoparticulate drugs. The drugs are physically trapped in assembled particles composed of different materials such as polymers (polymer micelles, dendrimers and polymer nanoparticles), lipids (liposomes), or organometallic compounds (carbon nanotubes). The first generation of anticancer nanoparticles approved by the FDA include liposomal drugs and polymer conjugates (Barenholz, 2012; Pérez-Herrero and Fernández-Medarde, 2015). However, certain products can be subjected to *in vivo* and clinical trials, while others remain limited to *in vitro* studies. Therefore, the effectiveness and safety of the nanoparticle drug constitute important assessed parameters.

As for the development of drugs with nanoparticle carriers, one example is Doxil^®^, the first nano-drug using liposomes approved by the FDA, which demonstrates the clinical performance advantages of doxorubicin in a variety of neoplastic conditions due to pharmacokinetics and the unique EPR-related biodistribution of liposomal doxorubicin (Barenholz, 2012; Wei et al., 2016). Doxil^®^ can reduce side effects, especially that of cardiac toxicity, and improve patients’ adherence and quality of life (Barenholz, 2012). Cisplatin is an anticancer drug prepared with a polymeric micelle through the formation of a metal-polymer complex between cisplatin and poly-(ethylene glycol)-poly(glutamic acid) block copolymers (Nishiyama et al., 2003; Uchino et al., 2005). These micelles are 28 nm in size with a very narrow distribution, demonstrate extremely extended blood circulation, and accumulate effectively in solid tumor models of Lewis lung carcinoma cells. However, because they undergo chemical synthesis, toxicity and scale-up production become major issues (Mizumura et al., 2001; Plummer et al., 2011; Mitchell et al., 2021). In addition, the development of Abraxane^®^, a paclitaxel-albumin-bound nanoparticle ∼130 nm in size, was approved by the FDA in 2005 for the treatment of metastatic breast cancer (Miele et al., 2009; Ma and Mumper, 2013). This formulation has been shown to have several advantages in terms of toxicity reduction compared to Taxol^®^. Moreover, the total dose can be given within 30 min without pre-treatment. However, the manner in which Abraxane^®^ can improve survival rates and overcome P-GP-mediated drug resistance remains unclear (Ma and Mumper, 2013).

Certain nanoparticles have been used in the delivery of UA as a cancer therapy including liposomes, polymeric nanoparticles, and polymeric micelles (Zaimy et al., 2017). However, at the time of writing, in contrast to other chemotherapy drugs such as Doxorubicin (Doxil^®^), Cisplatin, Paclitaxel (Taxol^®^), or Amphotericin B (Ambisome^®^), no review of the effectiveness and safety of several types of nanoparticles for the delivery of UA for cancer therapy has been conducted (Zaimy et al., 2017). This study aims to provide a literature review related to the anticancer effectiveness and safety of UA delivered with various types of nanoparticles based on pre-clinical and clinical trial results from existing research published between 2011 and 2021.

## Methods

### Article Selection Criteria

This study uses the scoping review method involving literature accessible through the PubMed, Sciencedirect, Scopus, and Google Scholar databases consisting of online research publications dating from 2011 to 2021. Clinical trial articles were sourced using the keywords"Clinical trial”, “Ursolic Acid”, “Cancer”, and “OR Nanoparticle Liposome”. As for the search for articles relating to *in vivo* studies, this employed the keywords “Pre-Clinical OR *in vivo* OR Animal”, Ursolic Acid”, “Cancer”, “Nanoparticle”. Within this study, several inclusion and exclusion criteria were applied to select and screen articles for review as shown in Table 1.

**TABLE 1:** The inclusion and exclusion criteria for article screening and selection.

Test parameters	Inclusion criteria	Exclusion criteria
Type of research	a) Randomized or non-randomized phases 1, 2, or 3 clinical trials	*a*) *In vitro* study
		*b*) *Ex vivo* study
	*b*) *In vivo* studies in animals	c) Review article
Intervention	a) Native UA as an active ingredient	a) Extracts containing UA and UA derivates
	b) Nanoparticles (lipids, polymers, hybrid nanoparticles as carriers)	b) Microparticles or other carrier systems more than 1,000 nm in size
	c) Administration routes comprise oral route in addition to intravenous, intraperitoneal, and intratumoral injection	c) Administration routes other than those meeting the inclusion criteria (topical, transdermal)
	d) Healthy patients and those suffering from all types of cancer (both individuals who have undergone surgery and those who have not)	
Comparison	a) No comparison with other drugs, only negative controls	-
	b) Comparison with other drugs	
Outcome	a) Primary efficacy outcomes (improved lifespan, enhanced survival rate, tumor growth inhibition)	-
	b) Secondary efficacy outcomes (e.g., blood parameters, no complaints); improvement in physical condition (body weight, tissue histopathology); clinical and non-clinical improvements	
	c) Toxicity (body weight, blood parameters, clinical parameters, non-clinical parameters, adverse events*,* organ histopathology)	
Types of Publications	a) Articles are written in English	The article is not written in English
	b) Not included as predatory journals	

### Evaluation of Physical Characteristics of UA Nanoparticles

Data analysis involved comparing the physical characteristics of different types of nanoparticles identified in the selected articles.

### Analysis of Particle Size

Particle size and particle size distribution produce significant impacts on drug loading variation, drug release, bioavailability, and efficacy (Kharia et al., 2012). In addition, particle sizes of 150 nm to 4.5 μm will be easily recognized by macrophages and dendritic cells during phagositosis (Koppolu and Zaharoff, 2013). Instruments used in nanoparticle size and morphology determination include Dynamic Light Scattering (DLS), Scanning Electron Microscopy (SEM), Transmission Electron Microscopy (TEM), and Atomic Force Microscopy (AFM).

### Analysis of ζ-Potential

Zeta potential is used to predict the stability of colloidal dispersion systems during storage. Generally, ζ-potential values above ± 30 mV resulted in more stable particles because the repulsing force between particles can prevent aggregation. The ζ-potential is affected by surfactants, polymers, the surface active agent component of nanoparticles, the presence of absorbing compounds, dispersed phase media, ionic strength, and pH (Kharia et al., 2012). The ζ-potential can be analyzed using the Electrophoretic Light Scattering (ELS) method (Miatmoko et al., 2017; Wang et al., 2017).

### Analysis of Encapsulation Efficiency

Entrapment Efficiency (EE%), or encapsulation efficiency, is defined as the portion of drugs encapsulated in nanoparticles. Free drugs that are not encapsulated in the drug delivery carriers or nanoparticles can be separated by means of centrifugation, dialysis, or gel chromatography. The concentration of entrapped and un-entrapped drugs can be determined through the use of instruments such as spectrophotometers or high-performance liquid chromatography (HPLC). The encapsulation efficiency percentage is calculated using the following equation:

$$EE\left(\%\right)=\frac{W_{T}-W_{U}}{W_{T}}\text{x }100\text{\%}$$

where, $W_{T}$ is the total weight of AU and $W_{U}$ is an un-entrapped UA weight (Poudel et al., 2020).

### Pharmacokinetic Evaluation of UA Nanoparticles

Plasma concentration versus time data was analyzed using non-compartmental methods. Peak plasma concentration $\left(C_{max\;}\right)\;$ and time-to-peak plasma concentrations ( $T_{max}$ ) were obtained through experimental observation. In elimination half-life $\left(t_{1/2\;}\right)$ calculated as 0.693/λz, λz is the slope of the terminal phase. In areas under the curve $\left({\text{AUC}}_{0-\text{t}}\right)$ of plasma concentration versus time from zero to infinity, ${\text{AUC}}_{0-\infty }$ is equivalent to the total area from time = 0 to the last measurable concentration time. The value is calculated using the linear trapezoidal method up to $C_{max\;}$ , log-trapezoidal method (until the last measured concentration), and extrapolated areas (Zhu et al., 2013). In this study, the analysis was conducted by comparing pharmacokinetic profiles from various studies contained in the articles reviewed.

### Evaluation of the Effectiveness of Ursolic Acid Nanoparticles

The analysis was conducted by comparing the results of efficacy studies including survival rate, tumor growth inhibition, tumor weight, and tumor volume, as well as tumor tissue histopathology extracted from reviewed articles.

### Cancerous Tissue Histopathology

The histological section of the liver was stained with haematoxylin and eosin (H&E) staining and subsequently compared to the histopathological appearance of each organ in the control and treatment groups (Melo et al., 2020). The microstructure and morphology of tissues were observed using a light microscope (Wang et al., 2017). Hematoxylin is a base dye that has an affinity for the acidic components of cells, primarily the nucleic acids contained in the nucleus, while eosin is an acidic dye that binds to the cell cytoplasm. As a result, H&E stained the core blue and cytoplasm orange-red (Slaoui and Fiette, 2011).

### Analysis of Relative Tumor Volume

In the articles, the size of the tumor is determined by means of a calliper, while its volume is calculated using the following equation:

$$\text{V}=0.5{\text{xy}}^{2}$$

where x is the longest and y the shortest diameter (Hattori et al., 2014; Yang et al., 2014; Wang et al., 2016; Miatmoko et al., 2017; Liu et al., 2021). In this study, the relative tumor volume is calculated using the following formulas:

$$relative\;tumor\;volume\;=\frac{{\text{V}}_{\text{T}}\text{ NC}}{{\text{V}}_{\text{T}}\text{ AUNP}}$$

or

$$\;\;relative\;tumor\;volume\;=\frac{{\text{V}}_{\text{T}}\text{ AU}}{{\text{V}}_{\text{T}}\text{ AUNP}}$$

where $\;{\text{V}}_{\text{T}}\text{ NC}$ is the volume of the negative control group tumor, ${\text{V}}_{\text{T}}\text{ AU}$ is the volume of the native UA treatment group tumor, and ${\text{V}}_{\text{T}}\text{ AU}$ NP is the volume of the UA nanoparticle treatment group tumor.

### Analysis of Relative Tumor Weight and Growth Inhibition Rate

The antitumor activity of nanoparticles is assessed through the tumor growth inhibition rate (IR) or tumor growth inhibition (TGI) at the experimental endpoint, calculated using the following equations:

$$\text{IR }\left(\text{\%}\right)\text{ or TGI }\left(\text{\%}\right)=\;\frac{{\text{W}}_{\text{T}}\text{ of negative control group}-{\text{W}}_{\text{T}}\text{ of treatment group}}{{\text{W}}_{\text{T}}\text{ of negative control group}}$$

where $W_{T}\;$ is the weight of the tumor (Wang et al., 2017; Shen et al., 2018; Fu et al., 2021). In this study, the relative tumor weight and relative inhibition rate were each calculated using the following formulas:

$$\text{Relative tumor weight}=\frac{{\text{W}}_{\text{T}}\text{ NC}}{{\text{W}}_{\text{T}}\text{ AUNP}}\text{or}\;\;\text{relative tumor weight}=\frac{{\text{W}}_{\text{T}}\text{ AU}}{{\text{W}}_{\text{T}}\text{ AUNP}}$$

where ${\text{W}}_{\text{T}}\text{ NC}$ is the weight of the negative control group tumor, ${\text{W}}_{\text{T}}\text{ AU}$ is the tumor weight of the native UA treatment group, and ${\text{W}}_{\text{T}}\text{ AUNP}$ is the volume of the UA nanoparticle treatment group tumor.

$$Relative\;inhibition\;rate\;=\frac{{\text{I}}_{\text{R}}\text{ AUNP}}{{\text{ I}}_{\text{R}}\text{ AU}}$$

where ${\text{I}}_{\text{R}}\text{ AUNP}$ is the tumor inhibition rate of UA nanoparticle treatment group, and ${\text{I}}_{\text{R}}\text{ AU}$ represents the tumor inhibition rate of the native UA treatment group.

### Analysis of Relative Survival Rate

Survival rates can be used as a standard assessment of effective therapy. The survival period is usually calculated from the date of diagnosis or commencement of the treatment period. The survival curve of each group was estimated using the Kaplan-Meier method with the average survival time difference being assessed by means of a log-rank test (Yang et al., 2014). This method involves non-parametric estimation of the survival function commonly used to describe the survival of a single population or compare the survival of two populations. The Kaplan-Meier estimate is one of the most effective statistical methods of measuring the probability of a patient’s survival observed during a post-treatment period (Etikan, 2017). In this study, the relative survival rate was calculated using the following formula:

$$\mathrm{Relative survival rate}=\frac{{\text{S}}_{\text{R}}\text{ AUNP}}{{\text{S}}_{\text{R}}\text{ NC}}$$

where $S_{R}\text{ AUNP}$ is the survival rate following the administration of UA nanoparticles and $S_{R}\text{ NC}$ is the survival rate of the negative controls.

### Evaluation of Toxicity of UA Nanoparticles in Pre-Clinical Trials

#### Weight Analysis

Weight loss represents a significant parameter of biological safety analysis or drug safety. The weight of the mice subjects is measured on the day of tumor inoculation and continues daily, or at least several times per week, during treatment. Each treatment group mouse is quantitatively weighed with the result being compared to that of a normal mouse in order to identify any significant difference between the two groups (Valcourt et al., 2020). In this study, the weight of the mice was calculated using the following formulas:

$$\text{Relative body weights }=\frac{{\text{W}}_{\text{B}}\text{ NC}}{{\text{ W}}_{\text{B}}\text{ AUNP}}\;\text{or }\;\text{Relative body weights }=\frac{{\text{W}}_{\text{B}}\text{ AU}}{{\text{ W}}_{\text{B}}\text{ AUNP}}$$

Where ${\text{W}}_{\text{B}}\text{ NC}$ is the mice body weight in the administration of negative control, ${\text{W}}_{\text{B}}\text{ AU}$ is the mice body weight in the administration of native UA, and ${\text{W}}_{\text{B}}\text{ AUNP}$ represents the mice body weight in the administration of UA nanoparticles.

#### Other Toxicity Analysis

Other toxicity data on the *in vivo* studies was derived by data recapitulation that included: tissue histopathology, increased levels of ALT and AST, and the amount of WBC as an indicator of hematological toxicity (Zhao et al., 2018; Li et al., 2019; Liu et al., 2021).

### Evaluation of AU-NP Toxicity in Clinical Trials

#### Analysis of Clinical Chemistry Data

Toxicity was evaluated in all subjects treated with at least one cycle of UA Liposome therapy. Hematological parameters (red blood cells, WBC, hemoglobin, ANC, and platelets), urinary routines (urine protein, glucose, erythrocytes, leukocytes, and urine bilirubin), and stool routines (stool erythrocytes and stool leukocytes) were evaluated. Blood biochemistry including ALT, AST, ALP, gamma-glutamyl transpeptidase (GGT) were further analyzed (Qian et al., 2015). In this study, the analysis was conducted by comparing clinical laboratory data (ALT, AST, GGT, DBIL, and TBIL) extracted from reviewed articles.

#### Analysis of Adverse Events

Adverse events are used to assess unintended events (AE) in healthy adult volunteers and patients with advanced solid tumor disease. In addition, the toxicity can be seen from the value of the maximum tolerated dose (MTD) used to determine the highest dose of the drug that can be administered without adverse events. The adverse events documented during the study were classified as mild, moderate, or severe based on the dosage (Valcourt et al., 2020). In this study, the analysis was conducted by comparing adverse events or side effects occurring in subjects who had received the treatment mentioned in reviewed articles.

## Results and Discussion

**FIGURE 1:**
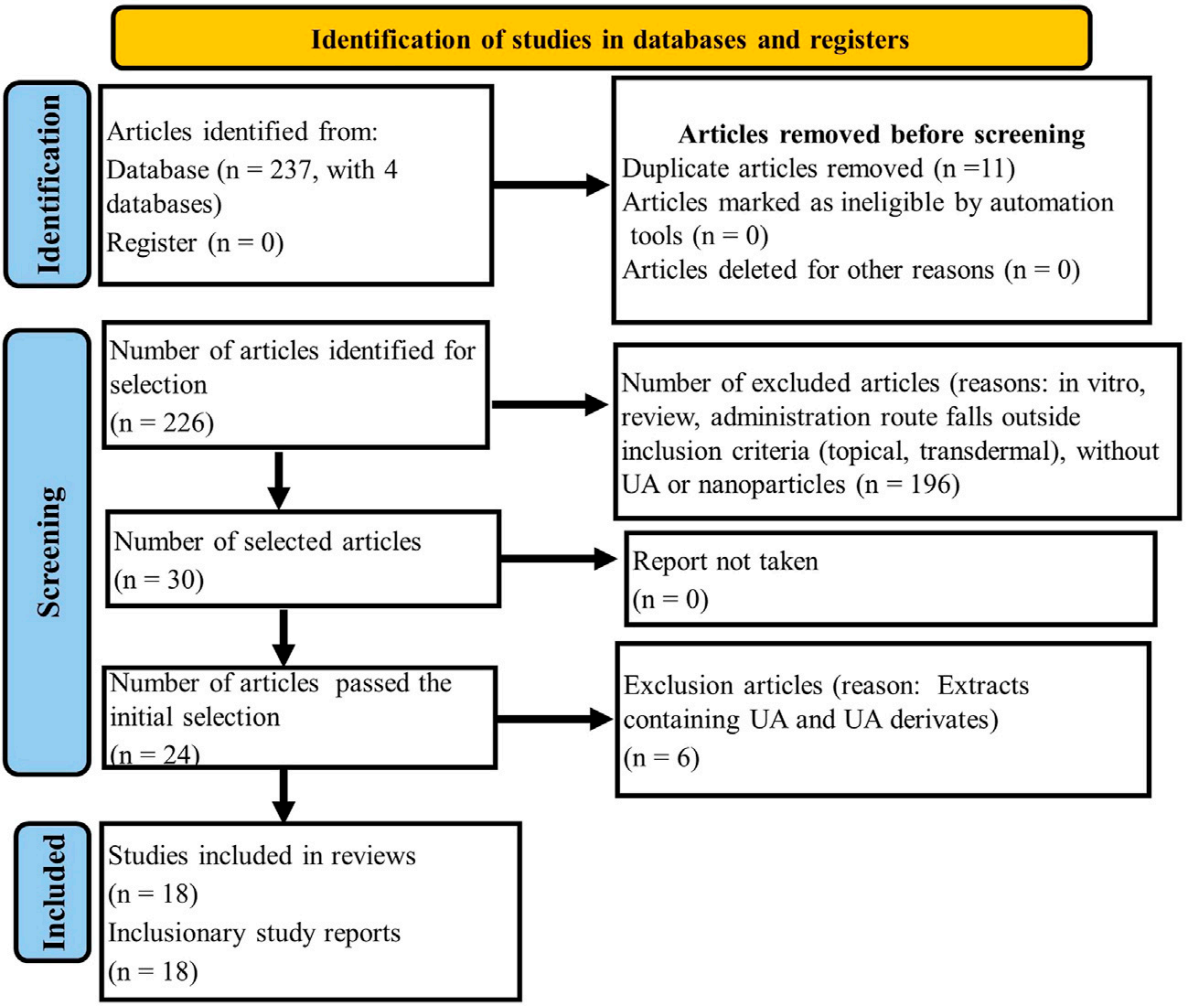
Flow chart of PRISMA method for article identification, screening, and selection.

This study provides a literature review focusing on the anticancer effectiveness and safety of UA delivered with various types of nanoparticles to increase its anticancer effects as confirmed by both pre-clinical and clinical trials. Literature searches of all four databases using pre-determined keywords identified 237 articles in the prescreening stage as can be seen in Figure 1. Of the total literature reviewed, duplication screening was conducted using the Mendeley application to produce a final body of 226 articles. Application of exclusion criteria resulting in a body of 196 selected articles which were subsequently subjected to inclusion criteria to produce a final total of 30. The initial selection process identified 24 articles which were subsequently reviewed, culminating in 18 which satisfied the inclusion criteria. The summary of reviewed articles is presented in Table 2.

**TABLE 2:** The summary of literature reviews for UA-loaded nanoparticles.

No	Code	Carrier type	Formula-tion	Type of research	Information research	Administra-tion route	Reference
1	Lipo A	Liposomes	Not Available	Phase I Clinical Trials	Safety Evaluation of Double Dose and Antitumor Activity of Ursolic Acid (UAL) Liposomes in Subjects with Advanced Solid Tumors including: Non-Hodgkin Lymphoma (24%), Hodgkin Lymphoma (24%), Renal Carcinoma (5%), Hepatocellular Carcinoma (5%), Breast Cancer (9%), Lung Cancer (9%), Other Cancers (19%)	Intravenous 4 h infusion at doses equivalent to 54, 74, and 96 mg UA/m^2^ for 14 consecutive days	Qian et al. (2015)
2	Lipo B	Liposomes	Not Available	Phase I Clinical Trials	Toxicity evaluation of a single dose of intravenous ursolic acid liposomes (UAL) in healthy adult volunteers and patients with advanced solid tumors including Non-Hodgkin Lymphoma, Hodgkin Lymphoma, Renal Carcinoma, and Hepatocellular Carcinoma	Intravenous (IV) route at doses equivalent to 11, 22, 37, 56, 74, and 96, and 130 mg UA/m^2^ administered as a 4 h infusion	Wang et al. (2013)
3	Lipo C	Liposomes	Not Available	Phase I Clinical Trials	Toxicity evaluation of Ursolic Acid Nanoliposome (UANL) in healthy volunteers and patients with advanced solid tumors including: Non-Hodgkin Lymphoma (50%), Hodgkin Lymphoma (12.5%), Gut Cancer (12.5%), Hepatocellular Carcinoma (25%)	Intravenous (IV) route at doses equivalent to 74 mg/m^2^ as a single dose, 98 mg/m^2^, and 74 mg/m^2^ as double doses daily for 14 days via a 4 h infusion	Zhu et al. (2013)
4	Lipo D	Liposomes	Hydrophobic components (PC, Chl, and UA) at a weight ratio of 2:1:0.5; ethanol injection method	Preclinical or *in vivo* study	Tumor inhibition activity and toxicity studies of UA-PLL-HA in SCC-7 tumor-induced mice	Intravenous (IV) at a dose of equivalent to 20 mg UA/Kg mouse for 5 times every 4 days	Poudel et al. (2020)
5	Lipo E	Liposomes	PEGylated UA Liposomes composed of SPC, CHOL, and UA at a weight ratio of 50:8:5, respectively; ethanol injection method	Preclinical or *in vivo* study	Tumor growth inhibition study and cytotoxicity of UA PEGylated liposomes in mice with U14 cervical carcinoma cells	Intragastric route at a dose of equivalent to 80 mg UA/kg mouse twice a day for a total of 14 days	Wang et al. (2016)
6	Lipo F	Liposomes	Liposomes composed of hydrophobic components (SPC, CHOL and UA) at a weight ratio of 0:6:5; ethanol injection method	Preclinical or *in vivo* study	Tumor growth inhibition and toxicity studies of CS-UA-L in mice with U14 cervical carcinoma cells	Intragastric route at a dose of equivalent to 80 mg UA/Kg mouse once a day for 14 days	Wang et al. (2017)
7	Lipo G	Liposomes	Lipids-UA (HSPC/Kolesterol/DSPE-PEG2000/UA = 90/0/5/5 and 90/0/5/10, (molar ratio); thin film hydration method	Preclinical or *in vivo* study	Tumor and growth inhibition study of UA-L in mice with 4T1 tumors (breast cancer)	Intravenous (IV) route at a dose of equivalent to 10 mg UA/kg mouse for 5 times every other day	Zhang et al. (2020)
8	Lipo H	Liposomes	Lipid components of FA-UA-L: DOTAP/CHOL/MPEG-DSPE2000/FA-PEG-CHEMS at a molar ratio of 40:55:4, 5:0, 5 (equal to weight ratio of 28; 21,3; 12,6, dan 2, 1 mg). The ratio of UA to lipid is 1:20 (w/w); thin film hydration method	Preclinical or *in vivo* study	Tumor growth inhibition and toxicity studies of FA-UA/siRNA-L in mice with human kB cells tumor	Intravenous (IV) injection with the dose of 4.5 mg/kg for UA and 170 μg/kg for siRNA for 5 times every other day	Li et al. (2019)
9	Lipo I	Liposomes	Lipid composition: HSPC/CHOL/mPEG-DSPE2000/FA-PEG-CHEMS at molar ratio 63:32:4.5:0.5 (equal to weights amount of 48, 12, 13.4, and 5 mg), respectively. The ratio of UA to lipids is 1:20 (w/w); thin film hydration method	Preclinical or *in vivo* study	Efficacy study of FTL-UA for tumor inhbition in mice with human KB tumor cells	Intravenous (IV) at a dose of equivalent to 4.5 mg UA/kg mouse for 5 times every other day, which is similar to 23 mg/kg or 98 mg/m^2^ drug administration	Yang et al. (2014)
10	Nano A	Nanospheres	Not available	Preclinical or *in vivo* study	Tumor growth inhibition and toxicity studies of HCPT @F-Pt-PU NPs in mice with H22 subcutaneous tumors (liver cancer)	Intravenous (IV) injection at a dose of equivalent to 10 mg UA/kg mouse for 5 times every 2 days	Liu et al. (2021)
11	Nano B	Nanospheres	NP composed of 32 mg chitosan, 10 mg UA, 30 mg EDC, and 8 mg NHS. The ratio of UA to lipids is 1:10 (w/w); overnight magnetic stirring method	Preclinical or *in vivo* study	Tumor inhibition study of CH-UA-NPs in mice with H22 subcutaneous tumors (liver cancer)	Oral administration at a dose of equivalent to 11 mg UA/Kg mouse once every 2 days for a total of 8 times	Jin et al. (2016a)
12	Nano C	Nanospheres	NP composed of 32 mg chitosan, 10 mg UA, 30 mg EDC, and 8 mg NHS. The ratio of UA to lipids is 1:10 (w/w); overnight magnetic stirring method	Preclinical or *in vivo* study	FA-CS-UA-NPs tumor inhibiting activity study in MCF-7 xenograft bearing models (breast cancer)	Intraperitoneal (IP) injection at a dose of equivalent to 12.5 mg UA/kg mouse once a day for 9 times	Jin et al. (2016b)
13	Nano D	Nanospheres	Not available	Preclinical or *in vivo* study	Tumor growth inhibition efficacy and toxicity studies of UA-LA-ICG NPs in tumor bearing mice by murine H22-hepatocarcinoma cells induced tumor xenograft models	Intravenous (IV) injection at a dose of 10 mg/kg of UA and 2.5 mg/kg of ICG with 5 min irradiation at 24 h post injection	Zhao et al. (2018)
14	Nano E	Nanospheres	Prepared by making 3 mg UA solution in ethanol (1 ml, 6,569 mM) in 10 ml of water. The ratio of UA and NPs was 1:10, respectively; solvent exchange preparation method	Preclinical or *in vivo* study	Tumor inhibition efficacy and toxicity studies of UA NPs in mice bearing A549 xenograft models (lung cancer)	Intravenous (IV) injection at a dose of 8 mg/kg of UA for 21 days	Fan et al. (2018)
15	Nano F	Nanospheres	Not available	Preclinical or *in vivo* study	Tumor inhibiting activity and toxicity studies of UA NPs in H22-induced mice (Hepatocellular carcinoma)	Intraperitoneal (IP) injection at a daily dose of 50 mg/kg of UA for 10 days	Zhang et al. (2015)
16	Nano G	Nanospheres	Self-assembly method of polymer deposition	Preclinical or *in vivo* study	Antitumor activity and toxicity studies of Pec-8PUH NPs in mice with 4T1 tumors (breast cancer)	Intravenous (IV) injection at a dose of 10 mg/kg of UA once every 2 days for 5 times	Liu et al. (2018)
17	Poli A	Polymeric Micelles	PM composed of UA (4 mg) and mPEG2000-PLA2000 (40 mg) at a weight ratio of 1:10; thin film dispersion method	Preclinical or *in vivo* study	Antitumor activity and toxicity studies of UA-PMs in H22-induced mice (Hepatocellular carcinoma)	Intraperitoneal (IP) injection at a dose of 50 mg/kg of UA every 2 days for 6 times	Zhou et al. (2019)
18	Poli B	Polymeric Micelles	Solvent evaporation method	Preclinical or *in vivo* study	Antitumor activity and toxicity studies of U-SS-M in tumor bearing MG-63/Osteosarcoma (OS)	Intravenous (IV) injection at a dose of 11 mg/kg of UA every 3 days for 5 times	Fu et al. (2021)

Notes: UAL, Ursolic Acid Liposome; UANL, Ursolic Acid Nanoliposome; UA-PLL-HA, Ursolic Acid-Poly-L-Lysine-Hyaluronic Acid; UA-PEGylated, Ursolic Acid-Polietilenglikolisasi; CH-UA-NPs, Chitosan-Ursolic Acid-Nanoparticles; CS-UA-L, Chitosan- Ursolic Acid-Liposome; CHOL/Chl, Cholesterol; DSPE-PEG2000, 1,2-distearoyl-sn-glycero-3-phosphoethanolamine-N [methoxy (poly- ethylene glycol) -2000]; DOTAP, 1, 2-dioleoyl-3-trimethylammonium-propane; EDC, Ethyl-(3-3-dimethylaminopropyl) carbondiimide hydrochloride; FA-CS-UA-NPs, Folate- Chitosan-Ursolic Acid-Nanoparticles; FA-PEG-CHEMS, Folate Polyethylene Glycol Cholesteryl hemisuccinate; FA-UA/siRNA-L, Folate- Ursolic Acid/Small Interfering RNA-Liposome; F-Pt-PU, Folic Acid-Pectin-Eight-Arm PEG-UA conjugate; FTL-UA, Folate Receptor Targeted Liposome-Ursolic Acid; HCPT @F-Pt-PU NPs, Hydroxycamptothecin @folic acid-pectin-eight-arm PEG-UA nanoparticle; HSPC, Hydrogenated Soybean Phosphatidyl Choline; mPEG2000-PLA2000, Monomethoxy Polyethylene Glycol 2000 Poly Lactic Acid 2000; MPEG-DSPE2000, Monomethoxy Polyethylene Glycol 2000-Distearoyl Phosphatidylethanolamine; NHS, N-Hydroxy-Succinimide; PC, Phosphatidylcholine; Pec-8PUH NPs, pectin-eight-arm polyethylene glycol-ursolic acid/hydrooxycampothecin nanoparticle; SPC, Soybean Phosphatidyl Choline; UA-NPs, Ursolic Acid- Nanoparticles; UA-LA-ICG NPs, Ursolic Acid- Lactobionic Acid -Indocyanine Green; UA-PMs, Ursolic Acid-Polymer Micelles; U-SS-M, Micelles assembled by PEG-SS-UA (polyethylene glycol using a disulfide bond)

The data extraction of the literature used can be seen in Table 2.

### Nanoparticle Characterization Results

**FIGURE 2:**
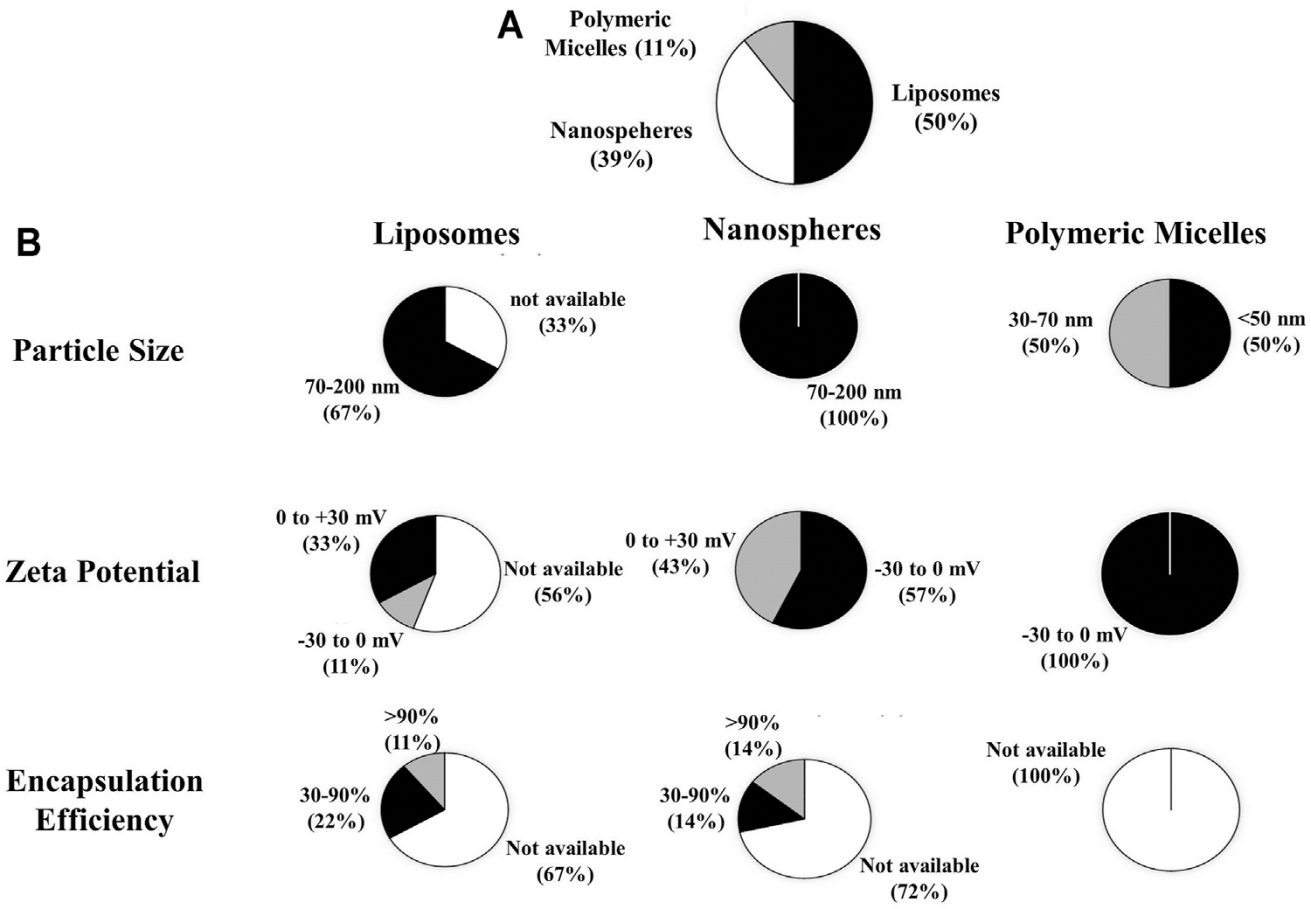
**(A)** Types of drug carrier extracted from the article review regarding the preclinical and clinical studies of nanoparticle use for UA delivery within cancer therapy, **(B)** the physical characteristics of UA-loaded nanoparticles including particle size, zeta potential, and efficiency of encapsulation.

From the review of the 18 articles, it was clear that three types of drug represent the most frequent delivery carriers of UA as an anticancer agent, namely; Liposome (50%), Nanosphere (39%) and Polymeric Misel (11%), (see Figure 2A).

According to the review results, several characterization parameters of liposomes, nanospheres, and polymeric micelles exist, including particle size, ζ-potential, and encapsulation efficiency (EE). From the data analysis of the 18 articles, the size of liposome particles was found to range from 70 to 200 nm (67%); nanosphere particle size to be between 70 and 200 nm (100%); and micelle polymeric particle size to be between 30 and 70 nm (50%). The ζ-potential of liposomes ranged from (−)30 to 0 mV (11%) and 0 to (+)30 mV (33%), while the nanosphere ζ-potentials were between (-)30 to 0 mV (57%), 0 to (+)30 mV (43%); and ζ liposomes of (−)30 to 0 mV (100%). For the EE of liposomes ≥90% (11%) and 30–90% (22%); EE nanospheres ≥90% (14%) and 30–90% (14%); as for EE polymeric micelles, these are not mentioned in the article, as presented in Figure 2B.

Characterization of liposome particle size is important because it affects the interaction of liposomes with target cells as well as the elimination, penetration and retention of drugs in the target sites (Monteiro et al., 2014). Phospholipids represent the main constituents of liposomal membranes and the use of lipid types and ratios within different preparation methods can affect the size of liposomes (Monteiro et al., 2014; Shaker et al., 2017). From Figure 2A it can be seen that liposomes prepared with ethanol injection and thin-film hydration methods have particle sizes ranging from 70 to 200 nm. This finding is in accordance with that of previous research arguing that, with the ethanol injection method, liposome could be generated as SUVs with diameters of 30–110 nm (Akbarzadeh et al., 2013; Monteiro et al., 2014), while with the adoption of thin film hydration methods, continued use of sonication or extrusion processes can produce liposomes as 25 nm to 1 μm-sized ULVs (Monteiro et al., 2014). Liposome size depends on that of the phospholipid molecule assembly whose average dimensions depend on their lipid composition, while it is supposed that the size of liposome particles increases slightly with a reduction in the molar ratio of HSPC/SPC in the range of 119–143 nm (Chen et al., 2006). On the other hand, liposomes made from DMPC, DSPC, and HSPC (at a weight ratio of 2:1 to cholesterol) experienced different increases in particle size, e.g., DMPC:Chol liposomes increased in size from 149 to 190 nm, DSPC:Chol expanded from 83 to 104 nm, and HSPC:Chol liposomes from 88 to 122 nm (Webb et al., 2019).

For nanospheres, particle size, which is greatly affected by lipid type, ranges from 70 to 200 nm. This is in accordance with a previous report stating that nanospheres have a diameter of 10–200 nm (Baldim et al., 2020). With regard to polymeric micelles, studies show that particle sizes ranging from 30–70 nm are affected by polymer types based on the characteristics of hydrophilic and hydrophobic block copolymers. This finding is in keeping with that of earlier research which reported that the size of polymeric micelle particles is determined by the ratio of hydrophobic and hydrophilic block chains and can produce particle sizes of ≤50 nm (Cabral et al., 2011). Increased targeting of drugs to cancer cells within the tumor tissues with the use of long-circulating polymeric micelles depends on the size of the micelle and the vascular permeability of the tumor tissues. In hypervascular tumors with highly permeable vascular structures, sub-100 nm polymeric micelles show no limits for drug extravasation and tumor penetration. In contrast, only micelles smaller than 50 nm can penetrate hipovascular tumors whose vascular permeability is poor (Cabral et al., 2011).

The zeta potentials which reflect the liposome surface charges were evaluated (Alves et al., 2017). Figure 2B shows that liposomes and nanospheres had zeta potentials ranging from (−30) to 0 mV and 0 to (+)30 mV, while those of polymeric micelles varied from (−30) to 0 mV. If the system has a strong negative or positive zeta potential the particles will tend to repulse each other and no aggregation occurs. Therefore, if the system has zeta potential >+30 mV or <−30 mV, then it can be considered stable (Laouini et al., 2012; Lunardi et al., 2021). The positive or negative charges measured in nanoparticles are highly dependent on lipid components. Analysis of the composition and intracellular delivery mechanisms confirmed that conventional liposomes had a relative neutral charge due to their neutral phospholipid composition such as HSPC and became negatively charged when added to cholesterol. pH sensitive liposomes contained a DOPE-like phospholipid component with CHEMS causing their negative charges; cationic liposomes had a cationic lipid composition such as DDAB, DOGS, DOTAP, DOTMA, DMRIE, DORIE with DOPE; Long-circulating liposomes (LCL) had a high T_C_ neutral lipid composition, cholesterol, added to approximately 5–10 mol % of PEG-DSPE rendering these liposomes stable when under protein opsonization (Sharma and Sharma, 1997; Gabizon et al., 2012).

The tendency of a drug to interact with polar or non-polar bonds and/or electrostatic interactions with lipid bilayer will determine whether it will be encapsulated into inner aqueous compartments or the lipid bilayer membrane, or whether it will be closely related to the polar head group of the bilayer membrane through electrostatic interactions. It will correlate to encapsulation efficiency (EE) or loading capacity, which is usually defined as a fraction of the percentage of the total encapsulated drug, in the bilayer membrane or aqueous intravesicular compartments or the matrix of nanoparticles (Kulkarni et al., 1995). UA has poor permeability and low water (Sun et al., 2020), thus causing possibly encapsulated within membrane bilayer of lipid vesicles. As can be seen in Figure 2B, the EE of liposomes and nanospheres ranges from 30% to ≥90%, while the EE of polymeric micelles is not mentioned. This suggests that drugs are successfully encapsulated in nanoparticles in order to increase the amount of drugs delivered to the target sites.

### Pharmacokinetic Data in Clinical Trials

From the review of pharmacokinetic data relating to clinical trials, it was found that in Lipo A the average ${\text{ t}}_{1/2}\;$ of UAL was 4.00–4.58 h, a low value of ${\text{t}}_{1/2}\;$ resulting in its rapid elimination from the blood circulation as shown by the contents of Table 3. This suggests that UAL does not accumulate in the body but must be infused repeatedly to ensure the steady plasma concentration of UA and further enhance its antitumor effect (Qian et al., 2015). In Lipo B, a linear relationship exists between C_max_ or $AUC_{0\to 24h\;}$ or $AUC_{0\to \infty \;}$ and increased doses of UAL, signifying that UAL has a linear pharmacokinetic profile (Wang et al., 2013). In Lipo C, after administration of a single IV dose, the total concentration of UA in all subjects experienced a two-fold decrease. On completion of IV infusion, the plasma concentration of UA rapidly decreases to one approximately ten times lower than the peak concentration after 2 hours. The pharmacokinetics profiles of UAL are linear and dosage proportional at a range of 37 mg/m^2^ to 98 mg/m^2^. No accumulation of UA was observed following repeated doses of UAL in eight patients after receiving continuous IV infusion 74 mg/m^2^ over a 14-day period (Zhu et al., 2013).

**TABLE 3:** Pharmacokinetic data from clinical trials of UA-loading nanoparticles.

Parameter	Lipo A	Lipo B			Lipo C			
Administration Route	Intravenous	Intravenous			Intravenous			
Dose (mg/m^2^)	74 (double dose)	37	74	98	37	74 (single dose)	98	74 (double dose)
${\text{t}}_{1/2}$ T1/2(hours)	4.58 ± 2.04	4.59 ± 2.44	4.46 ± 1.41	3.90 ± 2.08	4.59 ± 2.44	4.46 ± 1.41	3.90 ± 2.08	4.58 ± 2.04
${\text{V}}_{\text{d}}$ (L/m^2^)	NA	NA	NA	NA	58.7 ± 33.0	64.3 ± 17.9	55.4 ± 28.1	88.6 ± 31.8
CL (L/h/m^2^)	NA	8.65 ± 1.09	10.2 ± 1.46	9.94 ± 1.13	8.67 ± 1.07	10.20 ± 1.46	9.94 ± 1.13	14.40 ± 3.94
${\text{AUC}}_{0-\text{t}}$ (ng⋅h/mL)<	5,172 ± 1,136	4,213 ± 606	7,175 ± 999	9,696 ± 1,134	4,203 ± 588	7,175 ± 999	9,696 ± 1,134	5,172 ± 1,136
${\text{AUC}}_{0-\infty }$ (ng⋅h/mL)	5,498 ± 1,525	4,339 ± 574	7,418 ± 1,057	9,971 ± 1,144	4,329 ± 556	7,418 ± 1,057	9,971 ± 1,144	5,498 ± 1,525
${\text{MRT}}_{0-\text{t}}$ (hour)	NA	3.69 ± 0.36	3.93 ± 0.37	3.84 ± 0.34	3.69 ± 0.36	3.93 ± 0.37	3.84 ± 0.34	3.34 ± 0.55
${\text{MRT}}_{0-\infty }$ (hour)	NA	4.28 ± 0.91	4.56 ± 0.88	4.41 ± 0.95	4.29 ± 0.90	4.56 ± 0.88	4.41 ± 0.95	4.31 ± 1.89
${\text{C}}_{\text{max}}$ (ng/ml)	1,589 ± 635	1835 ± 438	2,865 ± 868	3,457 ± 856	1835 ± 438	2,865 ± 868	3,457 ± 856	1,589 ± 635
${\text{T}}_{\text{max}}$ (hour)	NA	4.03 ± 0.04	4.02 ± 0.04	4.0 ± 0.00	4.03 ± 0.04	4.02 ± 0.04	4.00 ± 0.00	3.00 ± 1.41

Notes: ${\text{t}}_{1/2}$ , half-life time; ${\text{V}}_{\text{d}}\;$ , distribution volume; CL, clearance; AUC, area under curve of concentration vs time; MRT, mean retention time; $\;{\text{C}}_{\text{max}}\;$ , maximum plasma concentration; ${\text{T}}_{\text{max}}\;$ , time required to reach maximum plasma concentration.

### Pharmacokinetic Data Review on *In Vivo* Studies

The pharmacokinetic data on *in vivo* studies of Lipo E shows that the highest plasma UA concentration in the PEGylated liposome treatment group was 19.87 ug/mL, which exceeded that of both the Ursolic Acid Liposomes (UAL) and Ursolic Acid (UA) solution groups. In addition, as seen from Table 4, PEG-modified liposomes have the longest ${\text{t}}_{1/2}$ , while ${\text{C}}_{\text{max}}\;$ and AUC in the bloodstream have similar trends. This suggests that PEGylated UA liposomes may extend the time required for the drug to circulate in the circulatory system and produce a slow release effect (Wang et al., 2016).

**TABLE 4:** Pharmacokinetic data summary of preclinical studies of nanoparticles containing UA.

Parameter	Lipo E	Nano A	Nano G	Poli B
Administration route	intragastric	intravenous	intravenous	intravenous
UA dose (mg/kg)	80	10	10	11
${\text{T}}_{1/2}\;$ (hour)	8.6	8.3 and 10	8.7	4.9 and 5.2
AUC (µg.h/ml)	134.061	NA	NA	NA
${\text{C}}_{\text{max}}\;$ (µg/ml)	19.87	NA	NA	NA
${\text{T}}_{\text{max}}\;$ (hour)	NA	80	80	48

Notes: ${\text{T}}_{1/2}$ , half-life time; AUC, area under curve of concentration versus time; ${\text{C}}_{\text{max}}\;$ , maximum plasma concentration; ${\text{ T}}_{\text{max}}\;$ , time required to reach maximum plasma concentration.

In Nano A, after administration of a hydrophobic drug, i.e., hydroxycamptothecin (HCPT), conjugated to folic acid-pectin-eight-arm PEG-UA (F-Pt-PU) the concentration of UA and HCPT in plasma decreases slowly, resulting in the longer circulation period of native UA, which may be due to the breaking of ester bonds between 8 arm-PEG and UA. The concentration of NP HCPT@F-Pt-PU in the bloodstream, still detectable at 80 h, is higher than that of native UA (7 h) and HCPT (8 h). The concentration of NP HCPT @F-Pt-PU in plasma is higher than that of np F-Pt-PU, possibly because the conjugation of HCPT into polymers increases the strength of the hydrophobic bonds in the particle cores, thereby reducing the hydrolysis rate of nanoparticles (Liu et al., 2021).

In Nano G, UA blood circulation in pectin-eight-arm polyethylene glycol-ursolic acid/hydrooxycampothecin nanoparticles (NP Pec-8PUH) at 80 h can be maintained at a higher concentration in plasma, while native UA and HCPT rapidly disappear from plasma. The half-life of UA blood circulation in NP Pec-8PUH is 8.7 h which is 7.3 times longer than in native UA (Liu et al., 2018).

In Poly B, polymeric drug conjugates are synthesized by conjugating UA into polyethylene glycol using disulfide bonds (U-SS-M), while UA is eliminated relatively slowly and maintained at high concentrations in plasma for up to 48 h after administration. U-SS-M exhibits a similar pattern of biodistribution and accumulates mainly in the liver and kidneys before being subsequently eliminated by these organs. In tumor tissue, the concentration of UA decreases over time, although the amount delivered by the polymer-drug conjugate gradually increases. The concentration of U-SS-M in tumor tissue is significantly higher than that of native UA at both 6 and 12 h after administration (Fu et al., 2021).

### Recapitulation of Pre-Clinical and Clinical Research Relating to UA Nanoparticles

**FIGURE 3:**
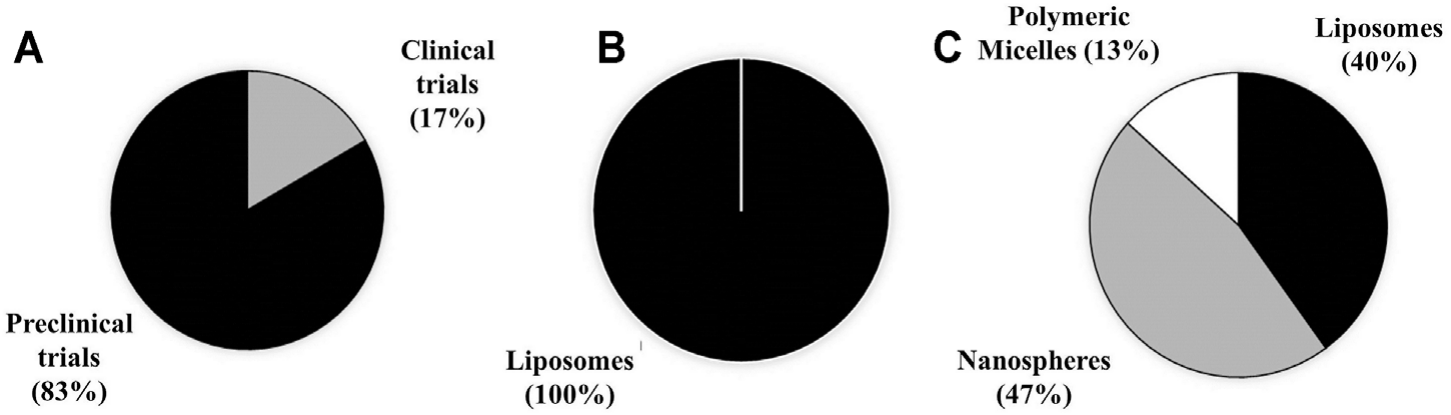
Research recapitulation of **(A)** clincial and preclinical studies, **(B)** types of nanoparticle use in clinical trials, **(C)** and pre-clinical trials.

The results indicate that the available articles which discuss pre-clinical/*in vivo* trials amounted of 83%, including the use of nanoparticle carrier types of nanospheres (47%), liposomes (40%), and polymeric micelles (13%). As for those that discussed clinical trials (17%), as seen in Figure 3A–C, these featured only the use of liposomes (100%). Clinical trials are still being conducted in phase 1, indicating that they remain at the stage of evaluating dose levels, acute toxicity, and drug distribution in humans (Valcourt et al., 2020).

### *In vivo* Anti-Cancer Efficacy of Nanoparticles Containing UA

#### Analysis of Tumor Tissue Histopathology

The anticancer effectiveness of nanoparticles containing UA compared to negative control and free UA are shown to have a significant effect on tumor growth inhibition as shown in Table 5.

**TABLE 5:** Tissue histopathology of liver cancer after administration of negative control, native UA and nanoparticles containing UA.

Code	Tissue histopathology		
	Negative control	Free UA	Nanoparticles containing UA
Lipo E	It features no hemorrhagic or necrosis phenomena and the cell is round or polygonal	Tumor cells and angiogenesis occur in native UA solution and conventional UA liposomes treatment groups, which become rare with slight necrosis	The tumor cells of the UA liposome with polyethylene glycols (PEGylated UA Liposome) group undergo severe necrosis, the nucleus/pulp ratio is significantly reduced, and apoptosis occurs due to a large number of scattered single tumor cells
Lipo F	The nucleus size and tumor cell shape are irregular. The tumor cells have clear cellular morphology and chromatin indicating that the tumor cells are growing quickly	A limited shrinkage and fragmentation of the nucleus indicates a low rate of tumor cell necrosis	Most tumor tissue cells in the group treated with Chitosan-Ursolic Acid-Liposomes (CS-UA-L) undergo apoptosis or necrosis, indicating good potential for killing cancer cells
Nano B	There are numerous sinusoids and small blood vessels filled with blood (indicated by the arrow) spreading through the hepatocellular carcinoma trabeculae	Not available	Several sinusoid liver or blood vessels can be observed in Chitosan-Ursolic Acid-Nanoparticle (CH-UA-NP) group with the exception of liver sinusoid dysplasia. Massive necrotic tissue can still be observed in hepatocellular carcinoma
Poli A	Tumor necrosis is undefined in the saline treatment group	Tumor cells and angiogenesis become rare with little necrosis	Most cancer cells in the high-dose Ursolic Acid-Polymer Micelles (UA-PMs) group at 100 mg/kg showed a high degree of H22 cell necrosis

##### Comparative Analysis of Tumor Growth Inhibition

**FIGURE 5:**
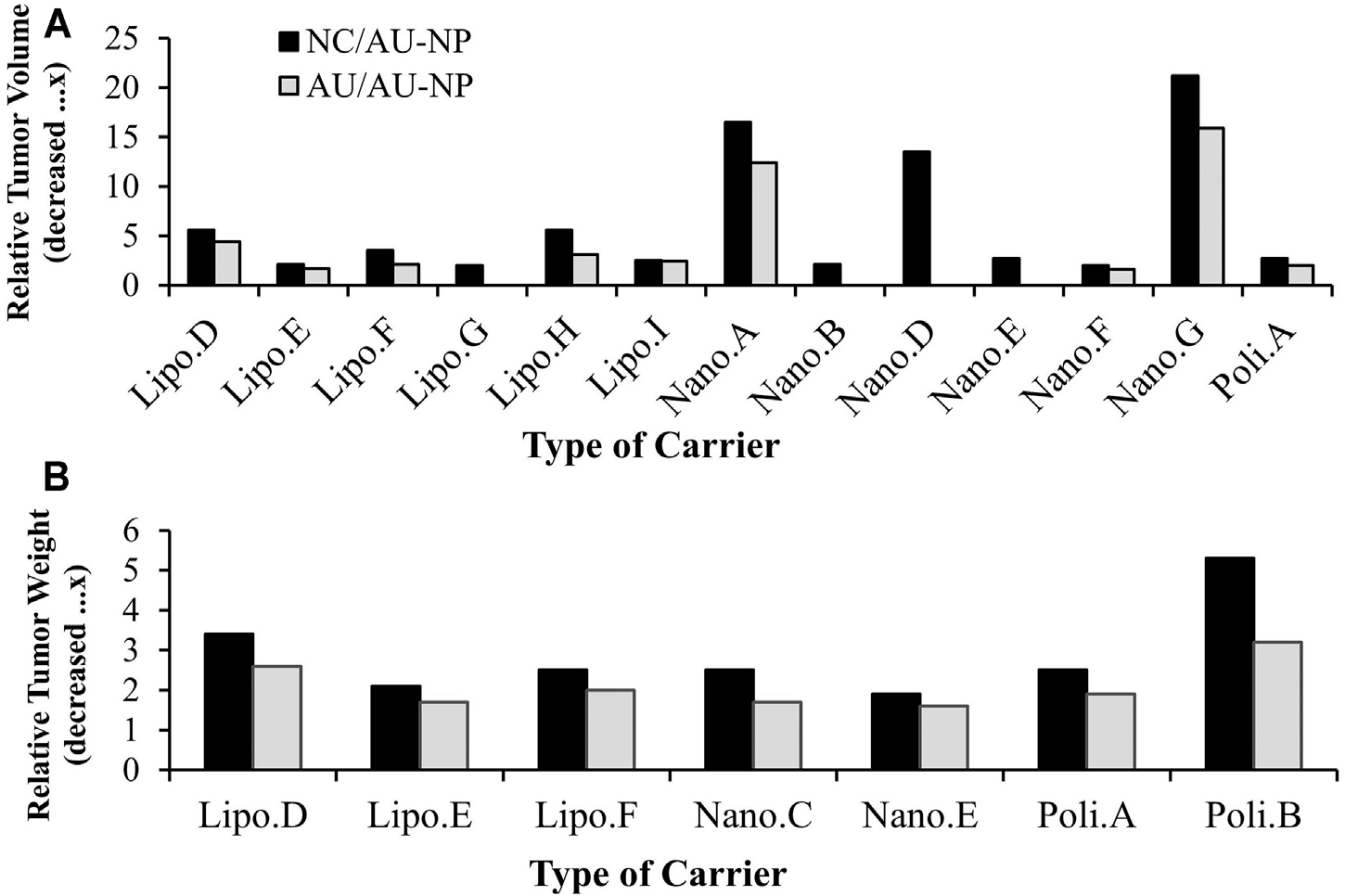
**(A)** Relative tumor volume in animal models treated with UA-loaded nanoparticles compared to negative control (black bars) and UA-free treatment groups (grey bars), **(B)** relative tumor tissue weight of animal models treated with UA-loaded nanoparticles compared to negative control (black bars) and native UA-treatment groups (grey bars).

The results indicate that the normal tumor volume when compared to administration of UA-loaded liposomes (Lipo D, E, F, G, H, I), nanospheres (Nano A, B, D, E, F, G) and polymeric micelles (Poly A) decreased in relative tumor volume by approximately 2.0–21.2 times. The tumor volume of native UA compared to the administration of UA liposomes (Lipo D, E, F, G, H, I), nanospheres (Nano A, D, E, F, G) and polymeric micelle (Poly A) showed a relative reduction in tumor volume of about 1.6–15.9 times lower than that of the native UA group, as presented in Figure 5A. This suggests that nanoparticles can improve UA effectiveness in inhibiting expansions in tumor volume.

The relative tumor weight analysis results relating to groups treated with UA liposomes (Lipo D, E, F), nanospheres (Nano C, E) and polymeric micelles (Poly A,B) indicated a relative reduction in tumor weight approximately 1.9–5.3 times that of the negative control group. Tumor weight in the native UA group compared to that of groups administered with UA liposomes (Lipo D, E, F), nanospheres (Nano C,E) and polymeric micelles (Poly A,B) showed a relative reduction of about 1.6-3.2x, as shown in Figure 5B. This suggests that nanoparticles may increase the effectiveness of UA in inhibiting tumor growth resulted in reduction of tumor weight.

**FIGURE 4:**
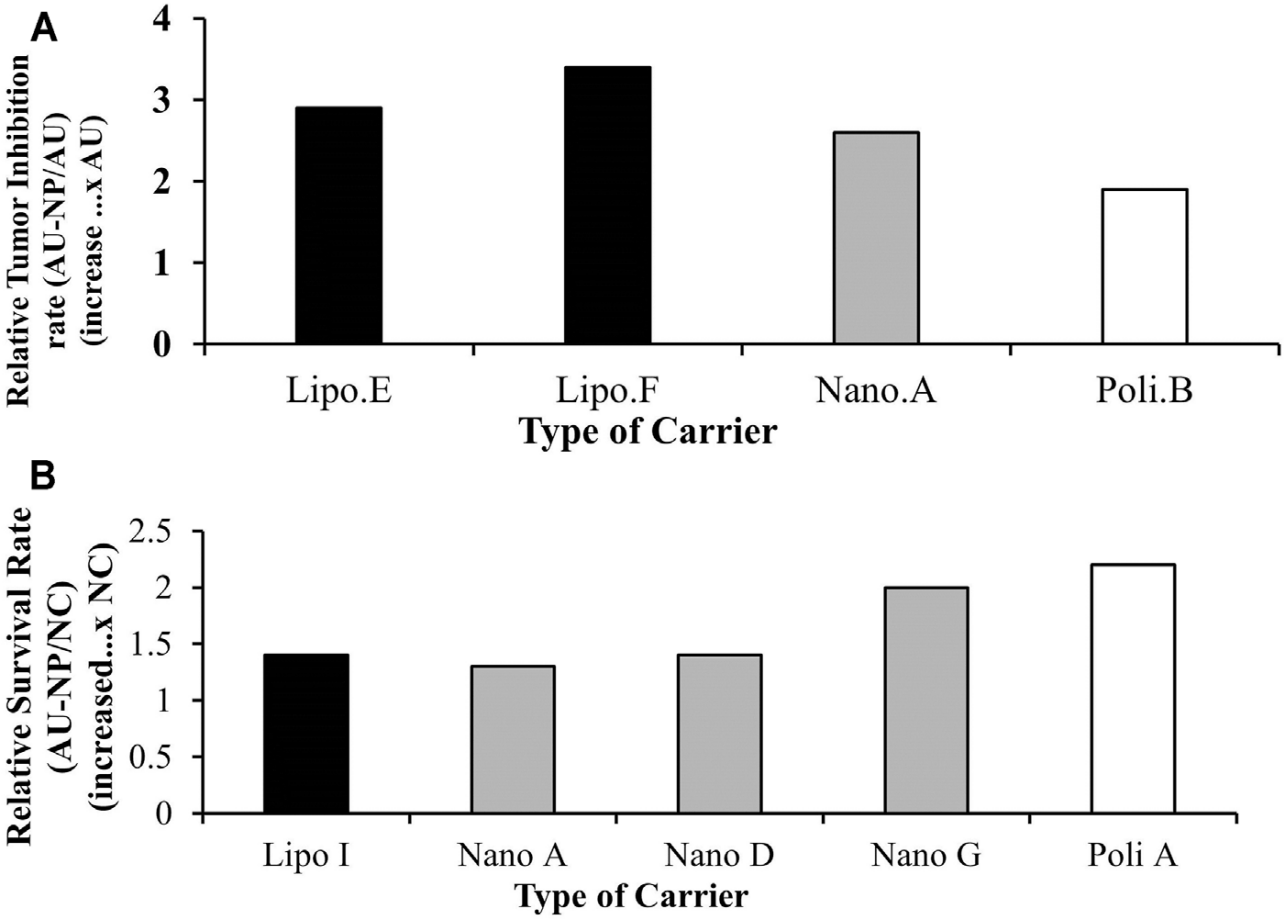
**(A)** Relative tumor growth inhibition rate of animal models treated with nanoparticles loading UA compared to native UA treatment groups, **(B)** Relative survival rate of animal models treated with UA-loaded nanoparticles compared to the negative control.

The relative inhibition rate analysis results indicate that the administration of UA liposomes (Lipo E, F), nanospheres (Nano A) and polymeric micelles (Poly B) produces an increase in the relative tumor inhibition rate of approximately 1.9-3.4x compared to the native UA group. Of the three types of drug carriers, liposomes (Lipo F) experienced the highest relative inhibition rate increase of 3.4x the native UA group, as seen in Figure 4A. This suggests that nanoparticles may increase the effectiveness of UA in inhibiting tumor growth.

##### Analysis of Survival Rate

Based on the results, the administration of liposomes (Lipo E, F), nanospheres (Nano A) and polymeric micelles (Poly B) produced an increase in the relative survival rate about 1.3-2.2x higher when compared to that of the negative control group, as seen in Figure 4B. This suggests that nanoparticles may increase the effectiveness of UA associated with improved survival rate.

The increased anti-tumor activity observed from the volume and weight of the tumor was associated with necrosis in the tumor tissues caused by large dose exposures of UA reaching cancer cells due to the increased permeability of small nanoparticles with high drug loading due to the EPR effect. Furthermore, the drug will be released into the extracellular and/or intracellular matrix. In the extracellular fluids, the drug will leak from nanoparticles and subsequently diffuse into cancer cells, while in intracellular fluids nanoparticles will experience endocytosis and the matrix will be destroyed in the endosome and release free drugs which then diffuse into the cytoplasm and nucleus subsequently causing cell necrosis. These results show that the use of nanoparticles as carriers within anticancer drug delivery can increase the *in vivo* survival rate.

Other studies have suggested that when nanoparticles such as liposomes interact with cells, drug delivery and diffusion into target cells can occur in several ways. Liposomes can penetrate the tumor tissue matrix resulting in degradation of carrier lipids by enzymes, such as lipase, or by mechanical strain inducing release of active substances into the extracellular fluid. This process induces drug diffusion into cell membranes culminating in cytoplasm and nucleus delivery. However, the latter process cannot easily be achieved by the use of hydrophilic drugs. Secondly, liposome membranes will fuse with those of the target cell leading to the release of liposomes directly into the cytoplasm. The third and most frequent method is that of receptor-mediated endocytosis. This process involves only vesicles with a maximum diameter of 150 nm and active substances demonstrating significant stability in such an acidic lysosome environment where liposomes are metabolized enzymatically. Phagocytosis may also ensue but involving large size nanoparticles affected by specialized immune system cells, such as macrophages, monocytes, and Kupffer cells. This process may eliminate the nanoparticles from the circulatory system (Bozzuto and Molinari, 2015).

The survival rate of liposomes is higher than that of other nanoparticles indicating the stability of the system in the blood circulation which ensures that the trapped drug is carried by the nanoparticles for further release into the cancer cells. In addition, because of the biomimetic property of liposome components that resemble phospholipid cell membranes it is easier for them to be absorbed by the cell.

### *In vivo* Toxicity Studies of Nanoparticles Containing UA

#### Pre-Clinical Toxicity Based on the Analysis of Relative Body Weight

From the results of relative body weight calculations contained in Table 6, no significant differences existed in the weight of the mice, proving that nanoparticle administration neither caused side effects nor produced symptoms of toxicity (Poudel et al., 2020; Liu et al., 2021; Fu et al., 2021). This result is also supported by other toxicity data presented in Table 7, which shows that there was no clear cell damage and no morphological changes in the major organs i.e. heart, liver, spleen, lungs, and kidneys. However, ALT, AST and WBC levels all decreased after administration of UA nanoparticles when compared to native UA (Zhang et al., 2015; Zhao et al., 2018). This suggested that UA nanoparticles do not cause serious toxicity, indeed, do not even produce toxicity. Rather, the effectt is mild and of short duration (Li et al., 2019).

**TABLE 6:** The relative body weight of animal models treated with UA-loaded nanoparticles compared to negative control and native UA-treatment groups.

Code	Toxicity
Lipo H	ALT and AST levels were significantly higher following an injection of FA-UA/siRNA-L compared to that of saline solution. The AST/ALT ratio of the FA-UA/siRNA-L group was significantly lower than that of the saline group. These results suggest that liver toxicity caused by liposomes produces mild, temporary liver toxicity
Nano A	The number of rat WBCs in the NP HCPT@F-Pt-PU treatment group increased more rapidly than in the native UA group which suggests that folate-targeted pectin delivery systems may prevent serious hematological toxicity
Nano D	There was no obvious cell damage or morphological changes in the major organs i.e., heart, liver, spleen, lungs, and kidneys in the NP UA-LA-ICG treatment group members compared to those of the negative control group
Nano E	ALT levels in mice treated with UA-NP were significantly lower than in the CCl4 group members, but there were no changes in the native UA- treatment group. In addition, AST levels in the UA-NP treatment group were also significantly lower compared to the CCl4 group and the native UA-treatment groups
Nano F	The native UA group experienced necropsy in the central section of the tumor tissue. These results partly suggest that native UA causes more toxicity than UA-NP. Meanwhile, H&E staining indicated that there were no obvious abnormalities or inflammatory lesions in any of the five organs, i.e., heart, liver, spleen, lungs, kidneys for the UA-NP treatment group when compared to their negative control and native UA counterparts
Nano G	Rats treated with the Pec-8PUH-NPs group did not experience any significant reduction in WBC counts as an indicator of hematotoxicity suggesting that the use of nanoparticles might prevent hematological toxicity

**TABLE 7:** Recapitulation of other preclinical toxicities.

Relative body weight		
Code	Results	
	NC/AU-NP	AU/AU-NP
Lipo D	Decreased by 1.2x normal value (Not significant)	Increased by 1.0x AU value (Not significant)
Lipo E	Decreased by 1.1x normal value (Not significant)	Increased by 1.0x AU value (Not significant)
Lipo F	Decreased by 1.2x normal value (Not significant)	Decreased by 1.0x AU value (Not significant)
Lipo G	There is no obvious difference	Not available
Lipo H	Decreased by 1.0x normal value (Not significant)	There is no obvious difference
Nano A	There is no obvious difference	There is no obvious difference
Nano D	There is no obvious difference	There is no obvious difference
Nano E	There is no obvious difference	There is no obvious difference
Nano F	Decreased by 1.0x normal value (Not significant)	Increased by 0.9x AU value (Not significant)
Nano G	There is no obvious difference	There is no obvious difference
Poli A	Decreased by 1.1x normal value (Not significant)	Decreased by 1.0x AU value (Not significant)
Poli B	There is no obvious difference	There is no obvious difference

### Safety Aspects of the Use of Nanoparticles Containing UA Based on the Clinical Trials

#### Toxicity Based on Clinical Laboratory Parameters

**FIGURE 6:**
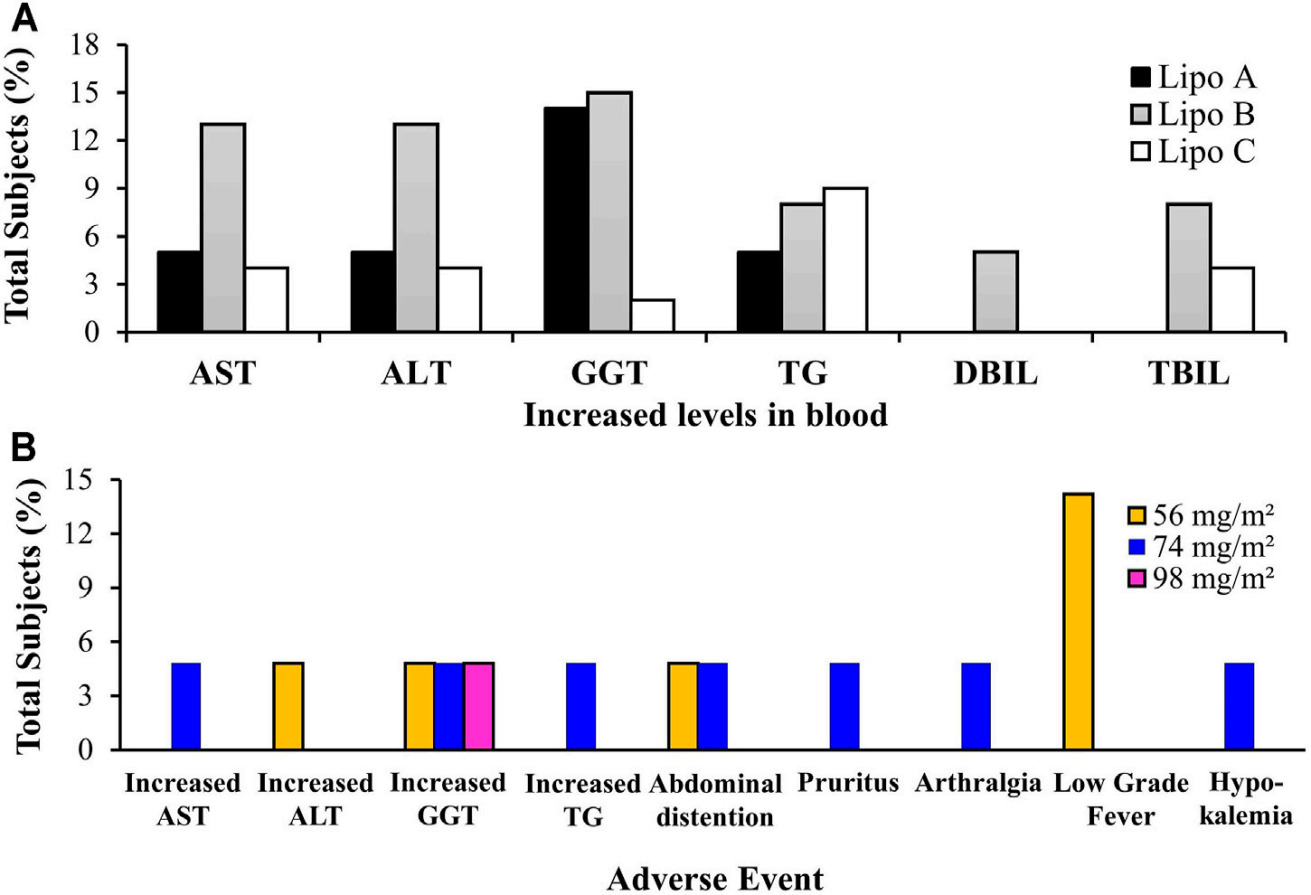
**(A)** Total subjects with increasing blood marker levels on clinical trials of Lipo A, Lipo B, and Lipo C, **(B)** Adverse events of Lipo A during phase I clinical trials. Notes: AST, Alanine Aminotransferase/SGPT (serum glutamic pyruvic transaminase); ALT, Aspartate Aminotransferase/SGOT (serum glutamic oxaloacetic transaminase); GGT, Gamma Glutamyl Transpeptidase; TG, Triglycerides; DBIL, Direct Bilirubin; TBIL, Total Bilirubin.

The content of the graphs contained in Figure 6A, confirm an increase in levels of AST, ALT, GGT, TG, DBIL, and TBIL after UA liposome (Lipo A, B, C) administration occurred. It can also be seen that Dose Limiting Toxicity (DLT) related to hepatotoxicity, which was monitored for substantial side effect parameters, was at a moderate level (Valcourt et al., 2020).

In Lipo A, an increase in levels of AST (5%), ALT (5%), GGT (14%), TG (5%) was observed in 21 subjects who received doses of 56, 74, and 98 mg/m^2^. In Lipo B, elevated levels of AST (13%), ALT (13%), GGT (15%), TG (8%), DBIL (5%) and TBIL (8%) were recorded in 39 subjects receiving doses of 11, 22, 37, 56, 74, 98, and 130 mg/m^2^. In Lipo C, there was an increase in AST (4%), ALT (4%), GGT (2%), TG (9%), and TBIL (4%) levels observed in 24 subjects who received doses of 74 mg/m^2^ as a single dose and 98 and 74 mg/m^2^ as multiple doses.

#### Clinical Toxicity Based on the Occurences of Adverse Events

According to the data contained in Figure 6B, three subjects (14%) treated with a dose of 56 mg/m^2^ of Lipo A experienced a mild fever but recovered after 2 hours without receiving treatment. Moreover, three subjects (14%) treated with sequential doses of 56, 74, and 98 mg/m^2^ of Lipo A experienced an increase in GGT, two subjects (10%) administered with doses of 56 and 74 mg/m^2^ of Lipo A experienced abdominal distension, and one patient (5%) experienced a rise in ALT levels. Other mild symptoms included increased AST and TG, pruritus, arthralgia, and hypokalemia. The most common adverse conditions included fever, increased GGT, and flatulence. These results indicated that a 4-h intravenous administration of Lipo A was tolerable and safe if a timetable of three doses per day for 14 consecutive days followed by a break lasting 7 days within each 21-days cycle was adhered to. Therefore, a 98 mg/m^2^ dose of Lipo A is the recommended dose for phase II trials (Qian et al., 2015).

**FIGURE 7:**
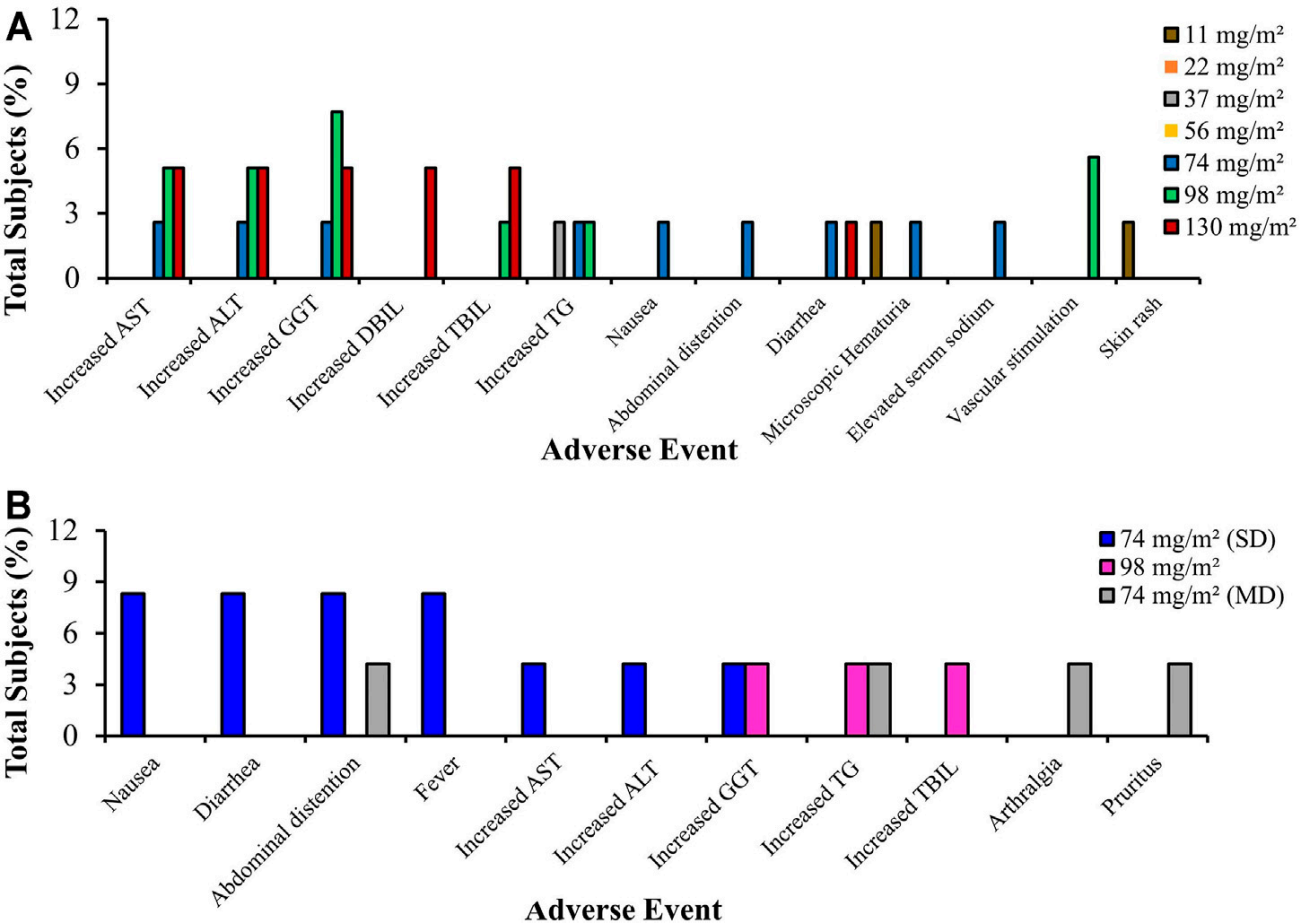
Total subjects with adverse events of **(A)** Lipo B, and **(B)** Lipo C in phase I clinical trials. AST, Alanine Aminotransferase/SGPT (serum glutamic pyruvic transaminase); ALT, Aspartate Aminotransferase/SGOT (serum glutamic oxaloacetic transaminase); GGT, Gamma Glutamyl Transpeptidase; DBIL, Direct Bilirubin; TBIL, Total Bilirubin; TG, Triglycerides

In addition, from the contents of Figure 7A, it can be seen that one patient treated with a 11 mg/m^2^ dose experienced a first degree skin rash which healed untreated after 3 days. In addition, two patients who had been administered with a 98 mg/m^2^ dose experienced vascular stimulation. First degree microscopic hematuria was observed in three subjects (7.7%) suffering from hepatoma malignancy who had received 11 doses of 11, 74, and 130 mg/m^2^, respectively. However, these side effects disappeared after 3 days without any treatment being administered. Elevated levels of AST, ALT, GGT, DBIL, and TBIL were observed in several subjects receiving doses of 74, 98, and 130 mg/m^2^. Dose Limiting Toxicity (DLT) resulted in hepatotoxicity: two subjects (5.1%) experienced an increase in AST, four subjects (10.3%) an increase in ALT, one subject (2.6%) an increase in GGT, and one subject (2.6%) an increase in DBIL. Diarrhea (2.6%) constitutes another DLT. Other drug-related side effects included nausea reported by one subject (2.6%), abdominal distension observed in another (2.6%), vascular stimulation occurred in two subjects (5.1%), while elevated TG was reported in three subjects (7.7%). Other reported adverse events included one subject (2.6%) suffering a skin rash and another (2.6%) experiencing higher serum sodium levels. At a dose of 74 mg/m^2^, one of six subjects experienced DLT, which is a form of non-hematological toxicity, including increased AST/ALT and diarrhea. At a dose of 98 mg/m^2^, one of the eleven subjects experienced DLT, i.e., non-hematological toxicity including increased ALT/GGT). At a dose of 130 mg/m^2^, two thirds of the subjects experienced DLT (increased ALT, AST, and DBIL). Therefore, the increased dosage was suspended and MTD was confirmed to be 98 mg/m^2^. Double administration of trial doses of UAL at recommended levels of 56, 74, and 98 mg/m^2^ was completed (Wang et al., 2013).

From the graph in Figure 7B, it is clear that all subjects in the study tolerated the Lipo C treatment. Most adverse events varying from mild to moderate related to Lipo C, which is Ursolic Acid Nanoliposome (UANL), were non-dose dependent. The most commonly observed adverse events included abdominal distension, nausea, and diarrhea. The adverse events after a 14-days continuous infusion of UANL comprised skin pruritus, arthrisgia, and increased triglycerides levels. UANL has minimal toxic effects. The limiting toxicity of UANL dose is hepatotoxicity. In this study, intravenous UANL infusions were well tolerated both by healthy volunteers and patients with advanced tumors (Zhu et al., 2013).

Based on the review analysis, only three articles which focused on liposomes as the drug carrier discussed clinical trials of UA. Although UA is classified as a BCS class IV drug, its permeability and solubility can be enhanced with the use of liposomes. It is related to the natural phase properties of the liposomal membrane that significantly affect permeability, aggregation, protein binding and liposome fusion. Membrane permeability largely depends on lipid components. Lipids that contain saturated chains or do not have carbon double bonds are more stable because they demonstrate greater resistance to oxidation. Lipid bilayer and liposome membranes possess a good lipid-packing order or gel phase below the lipid phase transition temperature (Tc), where the temperature is in a balanced proportion in the two phases. The fluidity of the lipid bilayer can be controlled by the selection and combined use of lipids, as the various Tcs depend on the length and origin sources (saturated or unsaturated) of fatty acid chains. For example, the incorporation of cholesterol at low concentrations into the lipid bilayer leads to increased *trans*-membrane permeability, where the incorporation of large amounts (>30 mol%) of cholesterol can reduce the transition phase and decrease membrane permeability at higher Tc temperatures (Corvera et al., 1992). Liposome permeability is related to the rate of solute diffusion through the lipid bilayer. The liposome membrane will achieve the highest permeability in the transition temperature phase, while its permeability is lower in gel form than in the fluid phase. The temperature of the bilayer phase transition is determined by the composition of the liposome. In the transition temperature phase, the permeability of liposomes to molecules such as protons and water increases (Koda et al., 2008; Yoshimoto et al., 2013; Hansen et al., 2015). In addition, the *in vivo* biodistribution and disposition of liposomes varies depending on the composition of the lipids, particle size, potential charge and degree of steric surface/hydration. In addition, the administration route may affect the *in vivo* disposition of liposomes. During intravenous administration, liposomes usually interact with serum proteins and are absorbed by RES cells, thus accumulating in the liver or spleen (Sercombe et al., 2015).

The development of nanoparticles for drug delivery, one of which is Doxil^®^ (Doxorubicin HCl liposome injection), the first nanoliposomal drug approved by FDA in 1995, was based on three principles: 1) prolonging drug circulation time and RES avoidance due to the PEGylation of nanoliposomes; 2) higher stable loading of doxorubicin driven by the transmembrane ammonium sulfate gradient which also allows the re-release of the drug in tumors; and 3) having lipid bilayer liposomes in a *“*liquid ordered*”* phase consisting of phosphatidylcholine with a high melting temperature ( ${\text{T}}_{\text{m}}$ = 53 °C), and which largely use cholesterol as a membrane stabilisator (Barenholz, 2012). In addition, various drug formulas in liposomes have received approval to be marketed and are widely used in clinical settings including Myocet*®* (Elan Pharmaceuticals Inc., Princeton, NJ, United States of America). This is an encapsulation of doxorubicin in liposomes (Swenson et al., 2001; Chastagner et al., 2015); Daunoxome^®^ (Gilead Sciences), daunorubicin formulated into liposomes (Forssen, 1997; Dawidczyk et al., 2014); Marqibo^®^ non‐PEGylated liposomal vincristine developed in 2012 as a therapy for various cancers including lymphoma, brain, leukemia, or melanoma (Silverman and Deitcher, 2012); Onivyde^®^ MM-398, which is a PEGylated liposomal irinotecan developed in 2015 as a drug to treat metastatic pancreatic cancer (Zhang, 2016), and many other forms of cancer (Ventola, 2017; Anselmo and Mitragotri, 2019). Various developments of the liposome delivery system indicated that liposomes possess non-toxic, flexible, biocompatible, and biodegradable properties that can enhance the therapeutic effects, safety, and efficacy of various anticancer drugs (Akbarzadeh et al., 2013).

As for the development of cisplatin therapy, which incorporates the use of an anticancer drug, this involves a polymeric micelle delivery system. Polymeric micelles were prepared through the formation of a metal-polymer complex between cisplatin (CDDP) and poly-(ethylene glycol)-poly(glutamic acid) block copolymers. Cisplatin polymeric micelles (CDDP/m) are 28 nm in size with the ability to distribute themselves through narrow spaces such as blood vessels in pancreatic tissue. These micelles undergo lengthy blood circulation and accumulate effectively in solid tumors of Lewis lung carcinoma cells. However, because they are produced synthetically, the toxicity and safety aspects as well as manufacturing production scale constitute extremely important issues (Plummer et al., 2011).

Abraxane^®^, a paclitaxel albumin-bound nanoparticle with a particle size of ∼130 nm which received FDA approval in 2005 for the treatment of metastatic breast cancer succesfully reduces toxicities in comparison to Taxol^®^. Moreover, it enables a complete dose to be administered within 30 min without the need for any pre-treatment. Nevertheless, the mechanism of Abraxane^®^ in improving survival rate and overcoming P-GP-mediated drug resistance remains unclear (Ma and Mumper, 2013).

The findings of this scoping review suggest that liposomes provide more comprehensive data than other forms of nanoparticles. This is demonstrated by the existence of *in vivo* studies of anticancer effectiveness assessed using several parameters such as increasing relative survival rate; more robust tumor growth inhibition (increasing relative inhibition rate, decreasing relative tumor weight, and reducing tumor volume); and improvements in tumor tissue histopathology. In addition, *in vivo* studies related to safety were also evaluated employing several parameters, i.e., weight loss, and other toxicity (lowering AST, ALT, and WBC), and well-tolerated toxicity by healthy volunteers and patients with advanced tumors.

There needs multi-faceted views of the use of nanoparticles for reviewing drug delivery. The components of the nanoparticle formulation would greatly affect the characteristics of the nanoparticles including particle size, potential charges, stealth and biomimetic properties, and others, which are closely related to drug delivery to cancer tissue, due to the Enhanced Permeation and Retention (EPR) effects. In addition, *in vivo* analysis of different types of cancer, where each type of cancer cell has different biological properties, also requires an in-depth study to provide data on supporting the effectiveness of drug delivery to target cancerous tissues. Moreover, the route of administration, dose, and frequency of drug administration related to the physicochemical properties and pharmacokinetic profile of the drug also greatly affect the systemic bioavailability and effective drug amount capable of reaching cancer tissue as the target of drug delivery. All these aspects provide important views for comprehensive study of the drug delivery system in cancer therapy.

## Conclusion

Based on the scoping review of the relevant literature, it can be concluded that UA loaded into nanoparticles is effective as a form of anticancer therapy. Pre-clinical trials confirm that it increases the relative survival rate*;* tumor resistance (increasing the relative inhibition rate, lowering the relative tumor weight, and decreasing tumor volume); and improves tumor tissue histopathology. In addition, UA-loaded nanoparticles have been proven safe for anticancer therapy based on the evaluation of weight loss and other toxicity (decreased AST/ALT). The results from the last 10-years analysis have indicated that, compared to nanospheres and polymeric micelles, liposomes have been assessed as more effective and safer during more comprehensive pre-clinical and clinical trials. This finding highlights the potential for liposomes to be further developed as a means of delivering UA as an anticancer therapy.
